# Supplementary figures and images for: From Cues to Signals: Evolution of Interspecific Communication via Aposematism and Mimicry in a Predator-Prey System
Source: PLoS One. 2014 Mar 10;9(3):e91783. doi: 10.1371/journal.pone.0091783 (PMC3948874; doi:10.1371/journal.pone.0091783)

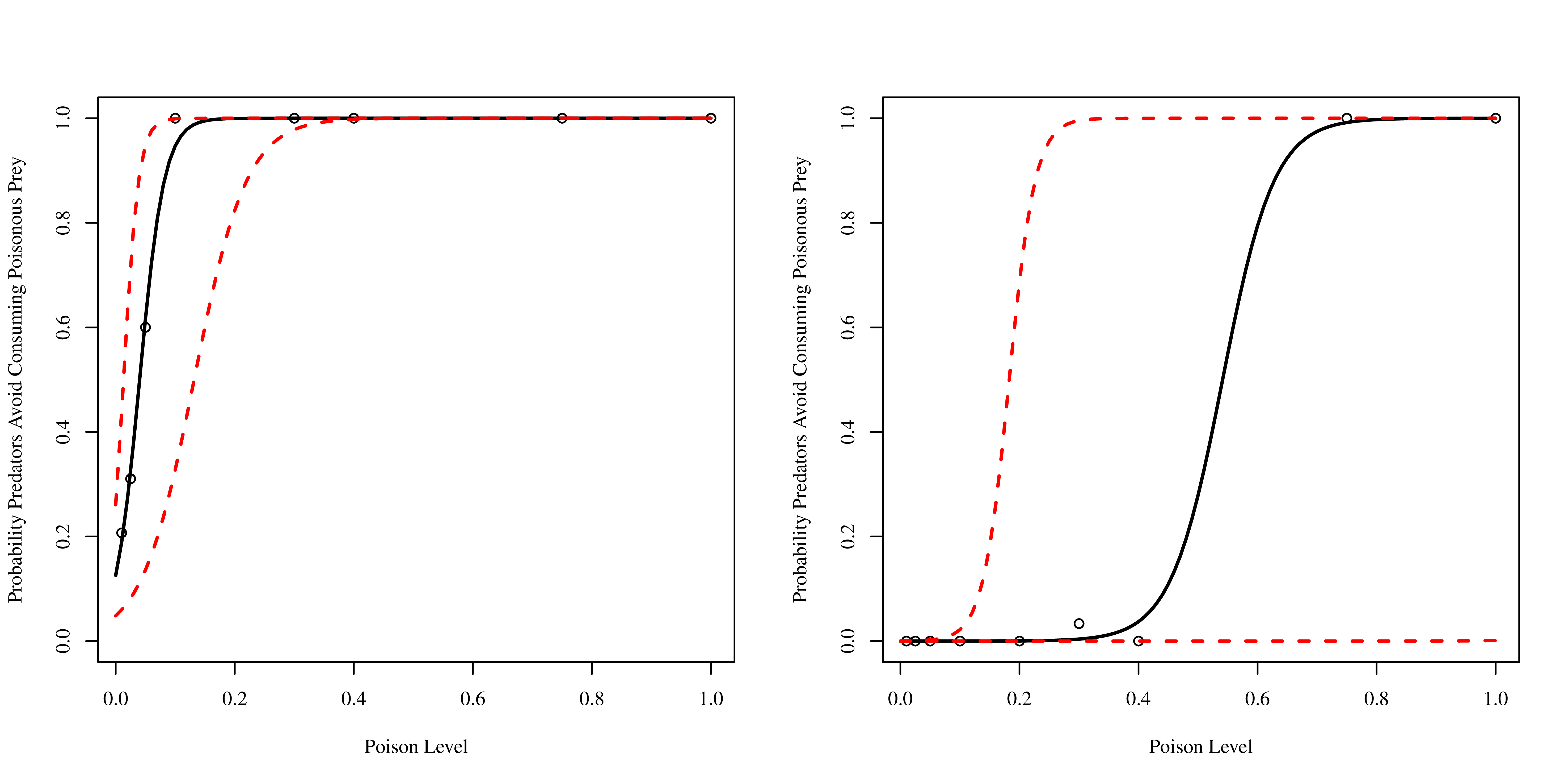

Supplement: Figure S1 — Prey to predator conversion efficiency shifts critical poison level thresholds, without altering overall patterns. Data shown represent fits from logistic regression models relating poison level to the probability that predator species will evolve to avoid consuming poisonous prey (based on proportion of evolved populations in which poison prey were no longer under predation pressure) when predators receive 10% (left) and 50% (right) of the value of their preys' consumed resources. Solid black line indicates the predicted probability. Red dashed lines represent the 95% bootstrap confidence intervals of the model. Circles indicate the observed values in our experiments. Due to low prey densities, in many populations, predators did not evolve into the systems when conversion efficiency was low and poison levels were high (only populations with at least 100 predators were considered here, n = 92 out of 270 for 10% and 270 out of 270 for 50%). (TIFF) [file pone.0091783.s001.tiff]

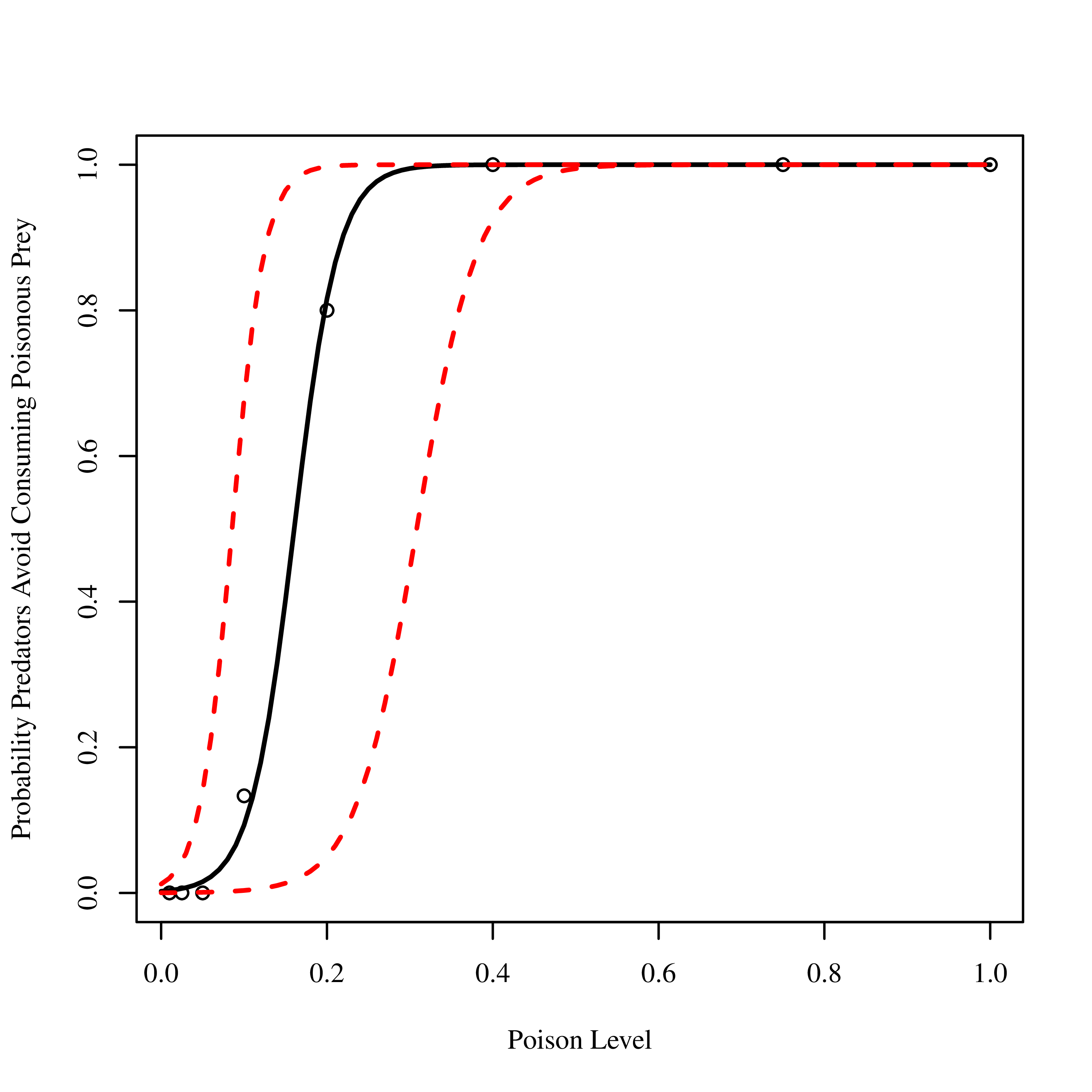

Supplement: Figure S2 — Exclusion of mimicry behaviors reduces poison levels needed to trigger the evolution of cue recognition. Data shown represent fits from logistic regression models relating poison level to the probability that predator species will evolve to avoid consuming poisonous prey (based on proportion of evolved populations in which poison prey were no longer under predation pressure) when mimic morphs were prevented from mimicking (compare to Fig. 1). Solid black line indicates the predicted probability. Red dashed lines represent the 95% bootstrap confidence intervals of the model. Circles indicate the observed values in our experiments. (TIFF) [file pone.0091783.s002.tiff]
